# Supplementary material for: STAT3 serine phosphorylation is required for TLR4 metabolic reprogramming and IL-1β expression
Source: Nat Commun. 2020 Jul 30;11:3816. doi: 10.1038/s41467-020-17669-5 (PMC7393113; doi:10.1038/s41467-020-17669-5)

Supplementary Information

STAT3 Serine phosphorylation is required for TLR4 metabolic reprogramming and IL-1β expression

Balic et al,

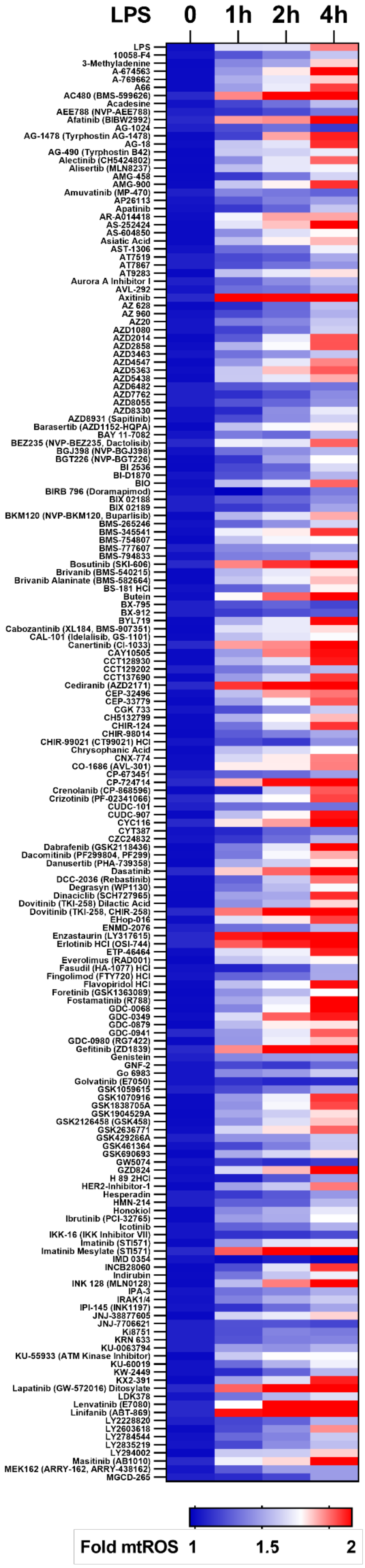
LPS
0
1h
2h
4h
LPS
10058-F4
3-Methyladenine
A-674563
A-769662
A66
AC480 (BMS-599626)
Acadesine
AEE788 (NVP-AEE788)
Afatinib (BIBW2992)
AG-1024
AG-1478 (Tyrphostin AG-1478)
AG-18
AG-490 (Tyrphostin B42)
Alectinib (CH5424802)
Alisertib (MLN8237)
AMG-458
AMG-900
Amuvatinib (MP-470)
AP26113
Apatinib
AR-A014418
AS-252424
AS-604850
Asiatic Acid
AST-1306
AT7519
AT7867
AT9283
Aurora A Inhibitor I
AVL-292
Axitinib
AZ 628
AZ 960
AZ20
AZD1080
AZD2014
AZD2858
AZD3463
AZD4547
AZD5363
AZD5438
AZD6482
AZD7762
AZD8055
AZD8330
AZD8931 (Sapitinib)
Barasertib (AZD1152-HQPA)
BAY 11-7082
BEZ235 (NVP-BEZ235, Dactolisib)
BGJ398 (NVP-BGJ398)
BGT226 (NVP-BGT226)
BI 2536
BI-D1870
BIO
BIRB 796 (Doramapimod)
BIX 02188
BIX 02189
BKM120 (NVP-BKM120, Buparlisib)
BMS-265246
BMS-345541
BMS-754807
BMS-777607
BMS-794833
Bosutinib (SKI-606)
Brivanib (BMS-540215)
Brivanib Alaninate (BMS-582664)
BS-181 HCl
Butein
BX-795
BX-912
BYL719
Cabozantinib (XL184, BMS-907351)
CAL-101 (Idelalisib, GS-1101)
Canertinib (CI-1033)
CAY10505
CCT128930
CCT129202
CCT137690
Cediranib (AZD2171)
CEP-32496
CEP-33779
CGK 733
CH5132799
CHIR-124
CHIR-98014
CHIR-99021 (CT99021) HCl
Chrysophanic Acid
CNX-774
CO-1686 (AVL-301)
CP-673451
CP-724714
Crenolanib (CP-868596)
Crizotinib (PF-02341066)
CUDC-101
CUDC-907
CYC116
CYT387
CZC24832
Dabrafenib (GSK2118436)
Dacomitinib (PF299804, PF299)
Danusertib (PHA-739358)
Dasatinib
DCC-2036 (Rebastinib)
Degrasyn (WP1130)
Dinaciclib (SCH727965)
Dovitinib (TKI-258) Dilactic Acid
Dovitinib (TKI-258, CHIR-258)
EHop-016
ENMD-2076
Enzastaurin (LY317615)
Erlotinib HCl (OSI-744)
ETP-46464
Everolimus (RAD001)
Fasudil (HA-1077) HCl
Fingolimod (FTY720) HCl
Flavopiridol HCl
Foretinib (GSK1363089)
Fostamatinib (R788)
GDC-0068
GDC-0349
GDC-0879
GDC-0941
GDC-0980 (RG7422)
Gefitinib (ZD1839)
Genistein
GNF-2
Go 6983
Golvatinib (E7050)
GSK1059615
GSK1070916
GSK1838705A
GSK1904529A
GSK2126458 (GSK458)
GSK2636771
GSK429286A
GSK461364
GSK690693
GW5074
GZD824
H 89 2HCl
HER2-Inhibitor-1
Hesperadin
HMN-214
Honokiol
Ibrutinib (PCI-32765)
Icotinib
IKK-16 (IKK Inhibitor VII)
Imatinib (STI571)
Imatinib Mesylate (STI571)
IMD 0354
INCB28060
Indirubin
INK 128 (MLN0128)
IPA-3
IRAK1/4
IPI-145 (INK1197)
JNJ-38877605
JNJ-7706621
Ki8751
KRN 633
KU-0063794
KU-55933 (ATM Kinase Inhibitor)
KU-60019
KW-2449
KX2-391
Lapatinib (GW-572016) Ditosylate
LDK378
Lenvatinib (E7080)
Linifanib (ABT-869)
LY2228820
LY2603618
LY2784544
LY2835219
LY294002
Masitinib (AB1010)
MEK162 (ARRY-162, ARRY-438162)
MGCD-265
Fold mtROS 1 1.5 2

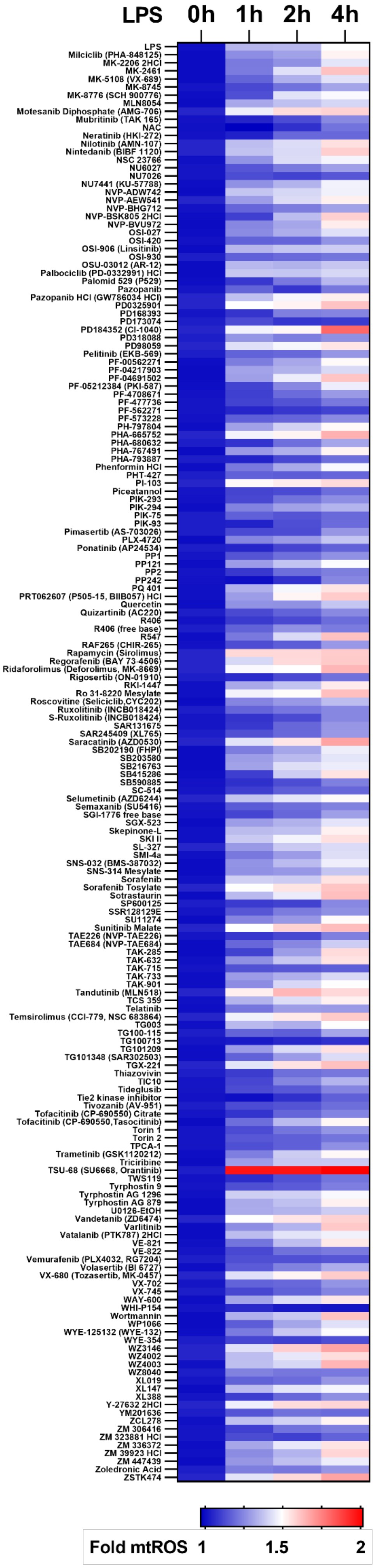
LPS
0h
1h
2h
4h
LPS
Milciclib (PHA-848125)
MK-2206 2HCl
MK-2461
MK-5108 (VX-689)
MK-8745
MK-8776 (SCH 900776)
MLN8054
Motesanib Diphosphate (AMG-706)
Mubritinib (TAK 165)
NAC
Neratinib (HKI-272)
Nilotinib (AMN-107)
Nintedanib (BIBF 1120)
NSC 23766
NU6027
NU7026
NU7441 (KU-57788)
NVP-ADW742
NVP-AEW541
NVP-BHG712
NVP-BSK805 2HCl
NVP-BVU972
OSI-027
OSI-420
OSI-906 (Linsitinib)
OSI-930
OSU-03012 (AR-12)
Palbociclib (PD-0332991) HCl
Palomid 529 (P529)
Pazopanib
Pazopanib HCl (GW786034 HCl)
PD0325901
PD168393
PD173074
PD184352 (CI-1040)
PD318088
PD98059
Pelitinib (EKB-569)
PF-00562271
PF-04217903
PF-04691502
PF-05212384 (PKI-587)
PF-4708671
PF-477736
PF-562271
PF-573228
PH-797804
PHA-665752
PHA-680632
PHA-767491
PHA-793887
Phenformin HCl
PHT-427
PI-103
Piceatannol
PIK-293
PIK-294
PIK-75
PIK-93
Pimasertib (AS-703026)
PLX-4720
Ponatinib (AP24534)
PP1
PP121
PP2
PP242
PQ 401
PRT062607 (P505-15, BIIB057) HCl
Quercetin
Quizartinib (AC220)
R406
R406 (free base)
R547
RAF265 (CHIR-265)
Rapamycin (Sirolimus)
Regorafenib (BAY 73-4506)
Ridaforolimus (Deforolimus, MK-8669)
Rigosertib (ON-01910)
RKI-1447
Ro 31-8220 Mesylate
Roscovitine (Seliciclib,CYC202)
Ruxolitinib (INCB018424)
S-Ruxolitinib (INCB018424)
SAR131675
SAR245409 (XL765)
Saracatinib (AZD0530)
SB202190 (FHPI)
SB203580
SB216763
SB415286
SB590885
SC-514
Selumetinib (AZD6244)
Semaxanib (SU5416)
SGI-1776 free base
SGX-523
Skepinone-L
SKI II
SL-327
SMI-4a
SNS-032 (BMS-387032)
SNS-314 Mesylate
Sorafenib
Sorafenib Tosylate
Sotrastaurin
SP600125
SSR128129E
SU11274
Sunitinib Malate
TAE226 (NVP-TAE226)
TAE684 (NVP-TAE684)
TAK-285
TAK-632
TAK-715
TAK-733
TAK-901
Tandutinib (MLN518)
TCS 359
Telatinib
Temsirolimus (CCI-779, NSC 683864)
TG003
TG100-115
TG100713
TG101209
TG101348 (SAR302503)
TGX-221
Thiazovivin
TIC10
Tideglusib
Tie2 kinase inhibitor
Tivozanib (AV-951)
Tofacitinib (CP-690550) Citrate
Tofacitinib (CP-690550,Tasocitinib)
Torin 1
Torin 2
TPCA-1
Trametinib (GSK1120212)
Triciribine
TSU-68 (SU6668, Orantinib)
TWS119
Tyrphostin 9
Tyrphostin AG 1296
Tyrphostin AG 879
U0126-EtOH
Vandetanib (ZD6474)
Varlitinib
Vatalanib (PTK787) 2HCl
VE-821
VE-822
Vemurafenib (PLX4032, RG7204)
Volasertib (BI 6727)
VX-680 (Tozasertib, MK-0457)
VX-702
VX-745
WAY-600
WHI-P154
Wortmannin
WP1066
WYE-125132 (WYE-132)
WYE-354
WZ3146
WZ4002
WZ4003
WZ8040
XL019
XL147
XL388
Y-27632 2HCl
YM201636
ZCL278
ZM 306416
ZM 323881 HCl
ZM 336372
ZM 39923 HCl
ZM 447439
Zoledronic Acid
ZSTK474
Fold mtROS 1 1.5 2

Supplementary Figure 1. Kinase inhibitors suppress LPS-induced mitochondrial ROS production, Related for Figure 3 and Supplementary Table 1

iBMDMs were pretreated with kinase inhibitors (500nM; see Supplementary Dataset 1) for 60 mins, and MitoSOX added 10 mins prior to challenge with LPS for indicated times. LPS-induced production of superoxide by mitochondria was analysed by measuring oxidized MitoSOX fluorescence at 580nm in a BMG ClarioStar. Data is represented as the means of 3 biological replicates/inhibitor (see Supplementary Dataset 2). Results are shown as fold increase compared to unstimulated control.

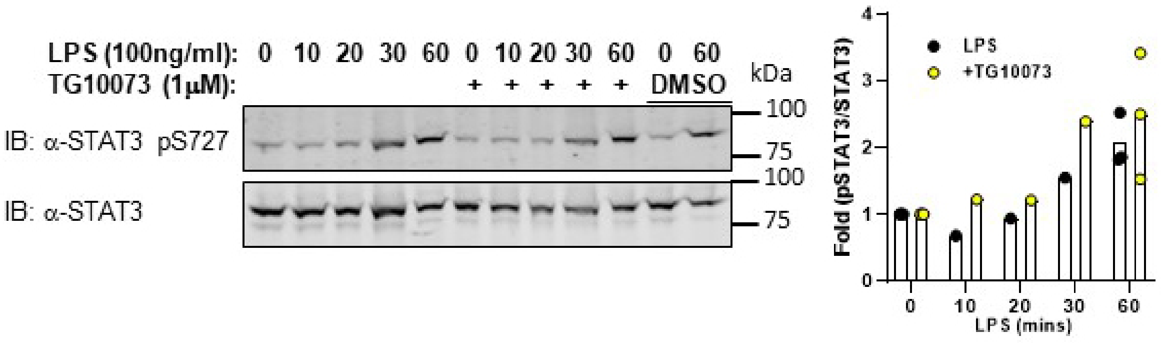

Supplementary Figure 2. PI3 kinase inhibition does not reduce LPS-induced STAT3 Ser727 phosphorylation.

The ability of PI3K to induce STAT3 Ser727 phosphorylation was determined by pretreating or not, BMDMs with the our highest ranked PI3K inhibitor TG10073 for 60 mins prior to LPS stimulation for indicated times. STAT3 Ser727 phosphorylation was determined in cell lysates by immunoblot with anti-STAT3 pS727 and anti-STAT3 antibodies. Densitometry was conducted using ImageJ software and presented as fold increase of STAT3 pS727 compared to total STAT3 protein as related to untreated or treated control for each sample (see Supplementary Dataset 3). DMSO alone was added as a solvent control. Data represents 2 biologically independent experiments for timepoints 10, 20 and 30mins post LPS; and 3 independent experiments for timepoints 0 and 60 mins post-LPS challenge.

Supplemental Figure 3 Full Figure Immunoblots

Uncropped immunoblots from Figure 1b

IP Samples: α-STAT3

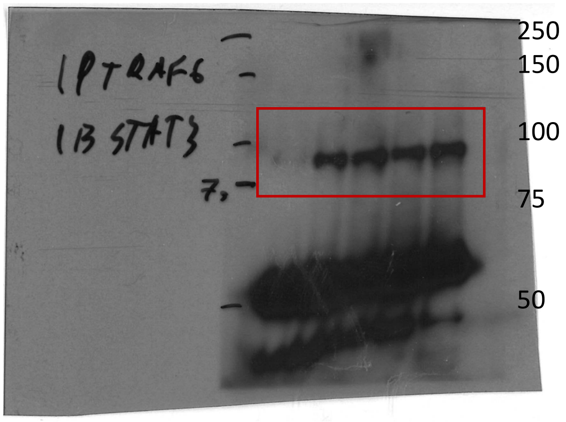

IB Lysates: α-STAT3

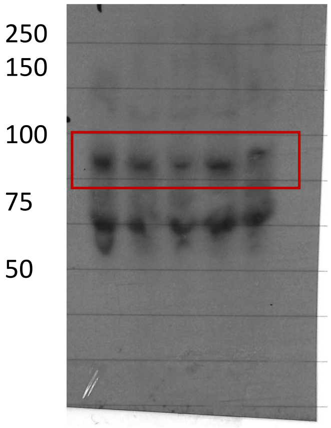

IP Samples: α-TRAF6

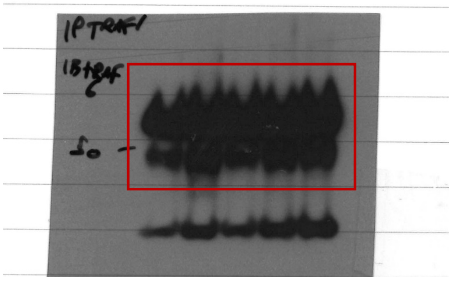

IB Lysates: α-TRAF6

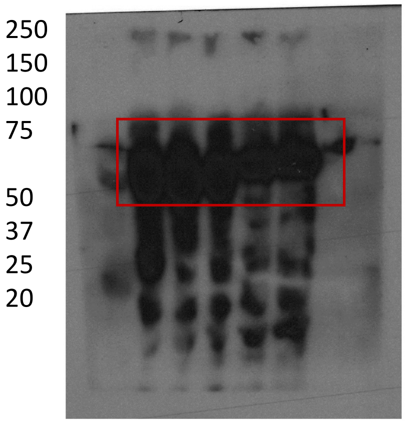

Uncropped immunoblots from Figure 1c

IP Sample: α-STAT3

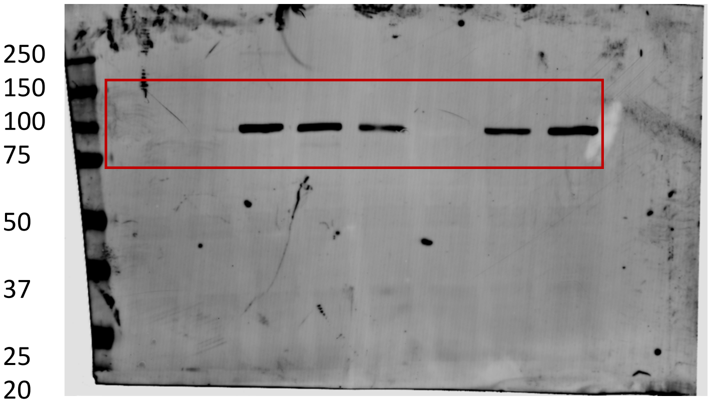

IB Lysates: α-STAT3

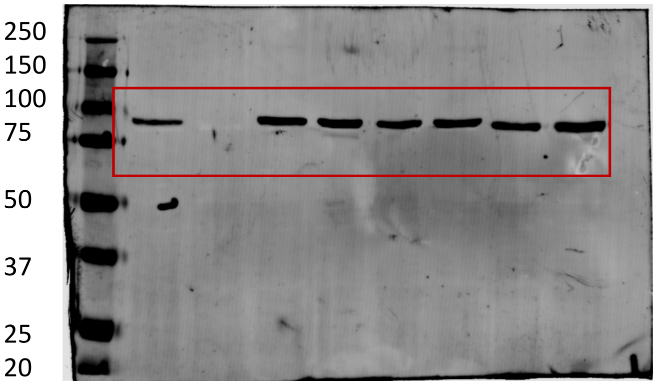

IP Sample: α-TRAF6

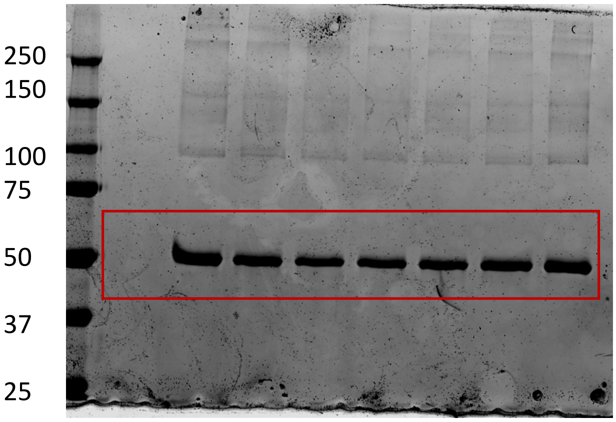

Uncropped immunoblots from Figure 2a

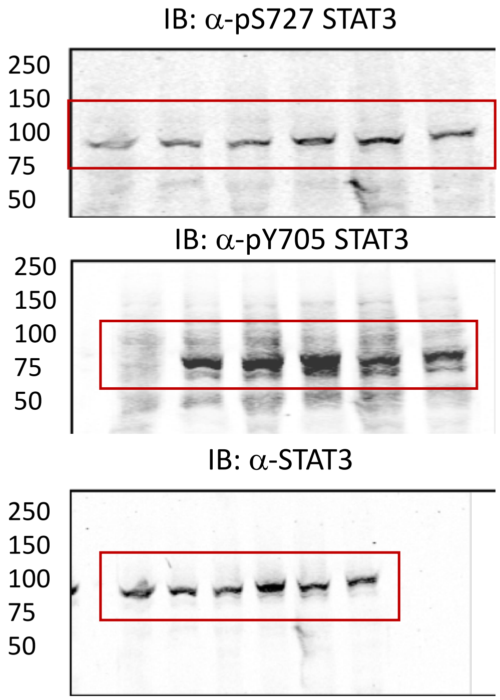

Uncropped immunoblots from Figure 2b

IB: α-pS727 STAT3

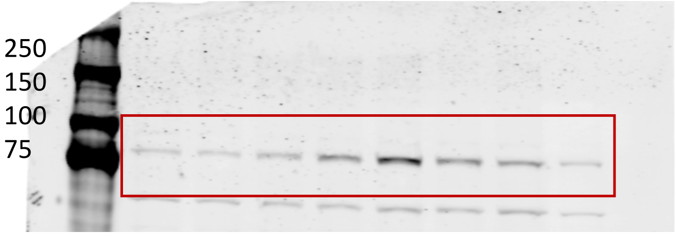

IB: α-pY705 STAT3

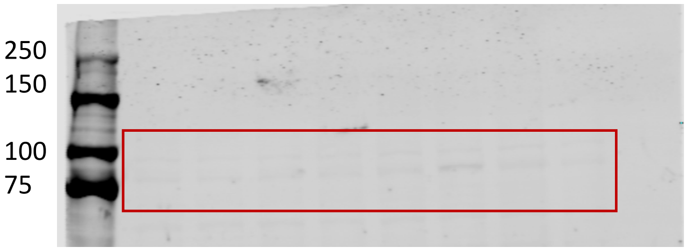

IB: α-STAT3

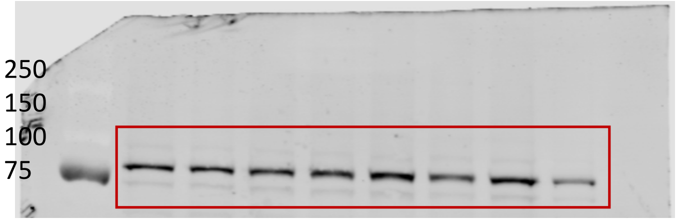

Uncropped immunoblots from Figure 2c

IB: α-pY705 STAT3

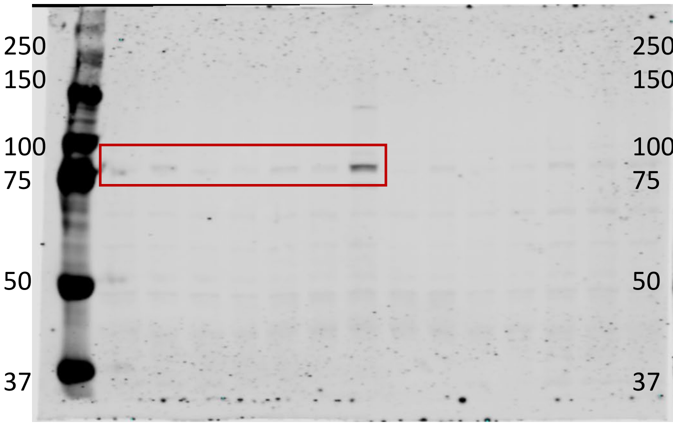

IB: α-pS727 STAT3

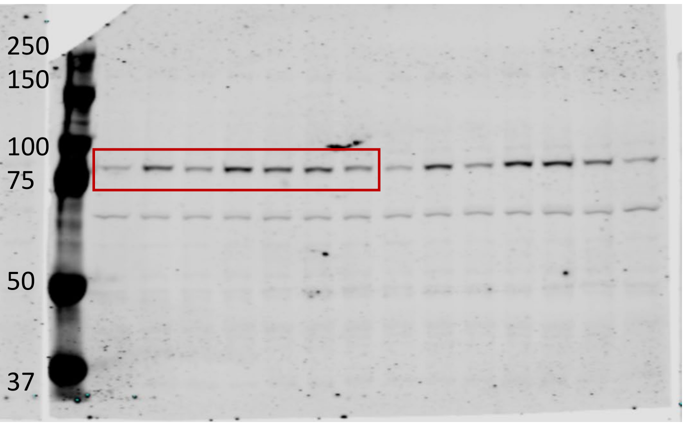

IB: α-STAT3

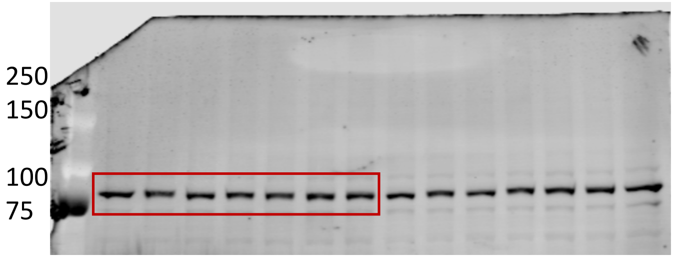

Uncropped immunoblots from Figure 2d

IB: α-pS727 STAT3

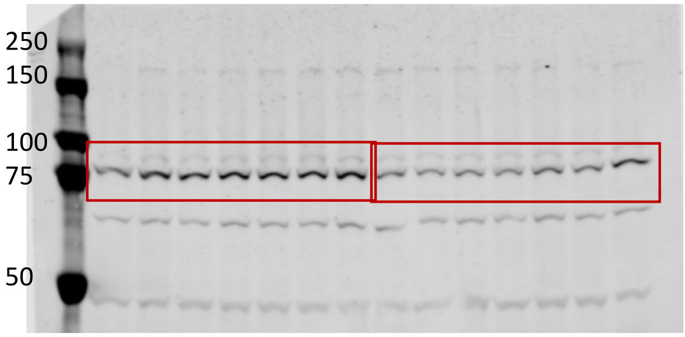

IB: α-STAT3

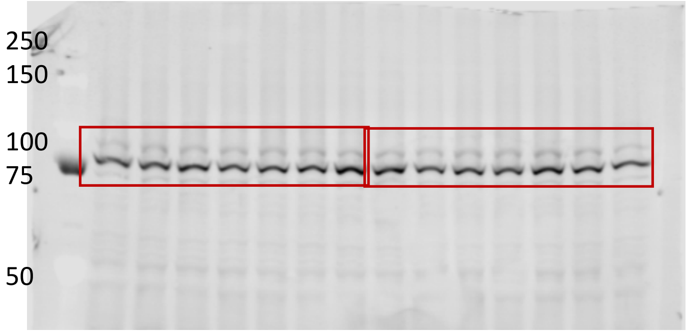

Uncropped immunoblots from Figure 2e

IB: α-pS727 STAT3

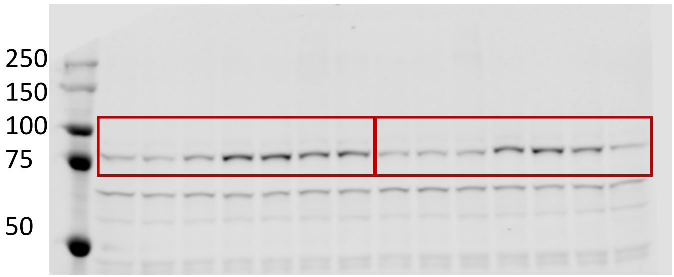

IB: α-STAT3

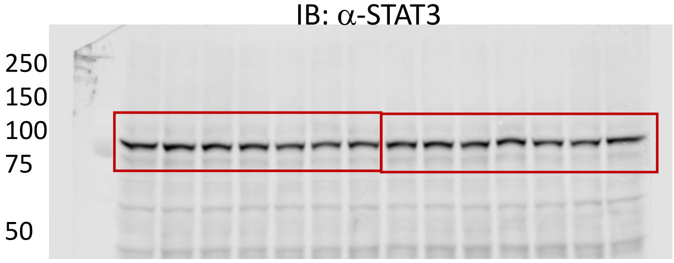

Uncropped immunoblots from Figure 2f

IB: α-STAT3 IB: α-pS727 STAT3

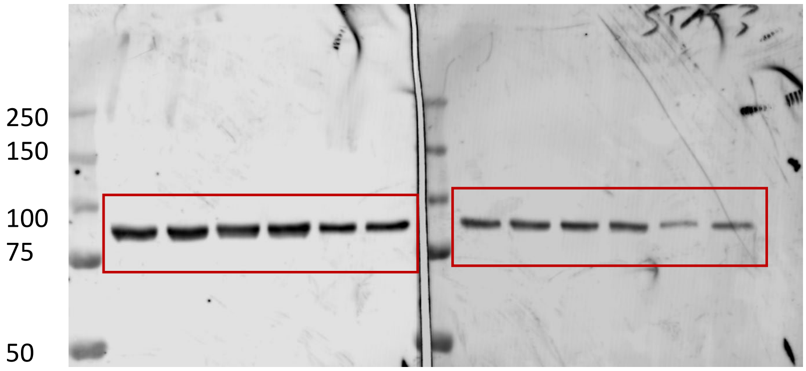

IB: α-VDAC

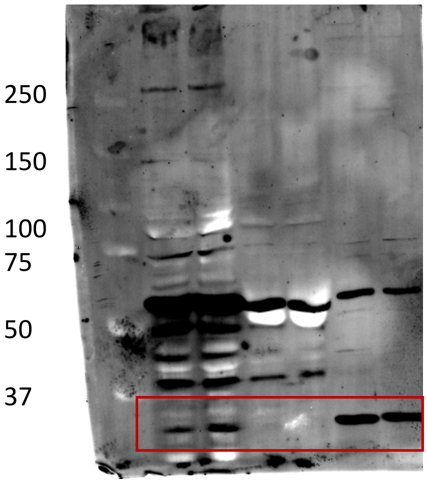

IB: α-ERK1/2

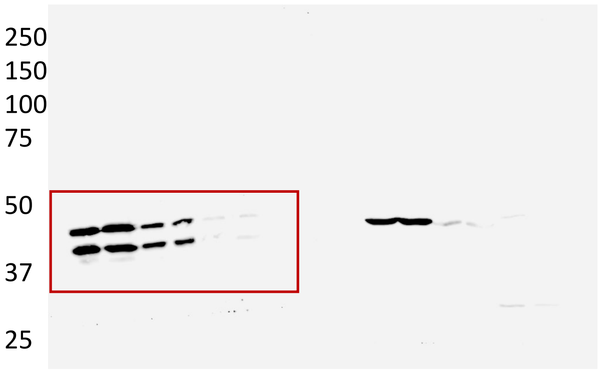

IB: α-Lamin A/C

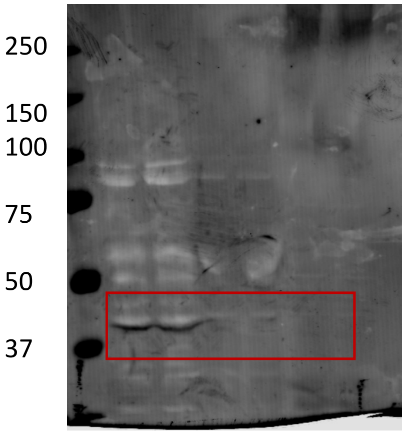

Uncropped immunoblots from Figure 3b

IB: α-TBK-1

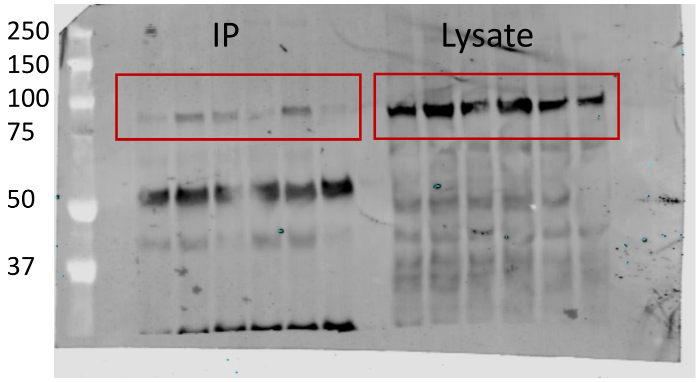

IB: α-pTBK-1

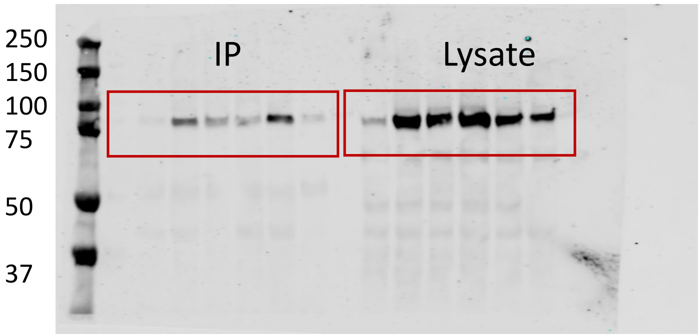

IB: α-TRAF6

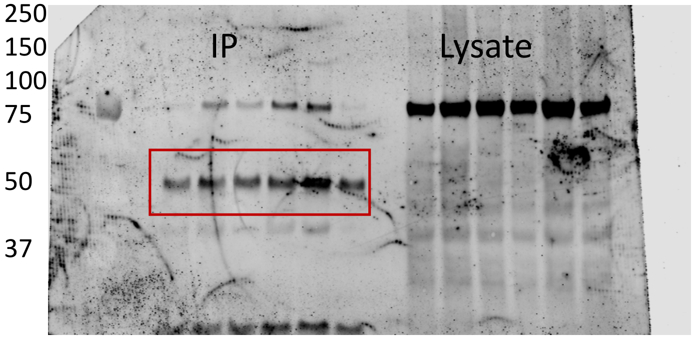

Uncropped immunoblots from Figure 3c

IB: α-pTBK-1

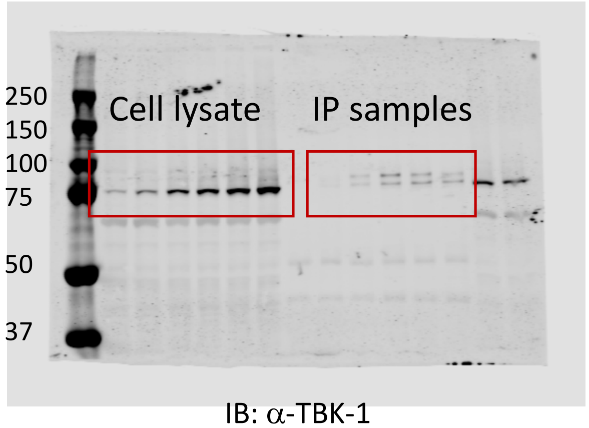

IB: α-TBK-1

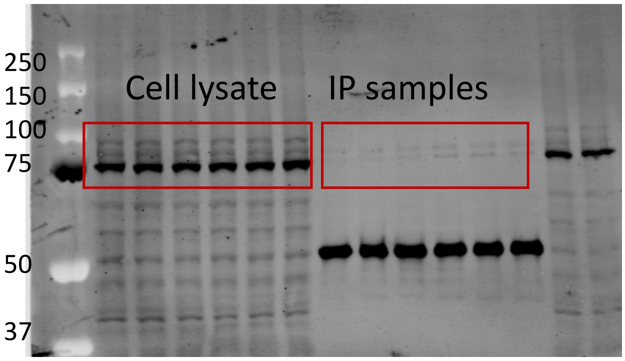

IB: α-STAT3

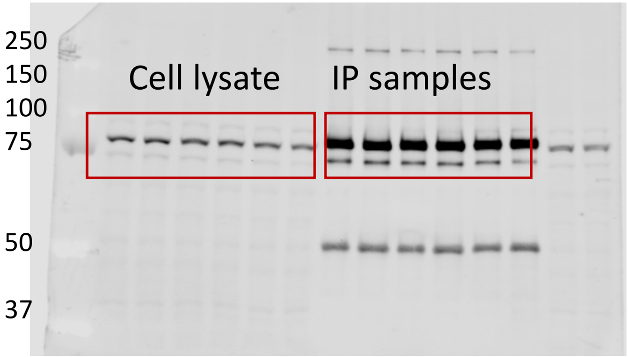

Uncropped immunoblots from Figure 3d

IB: α-pSer727 STAT3

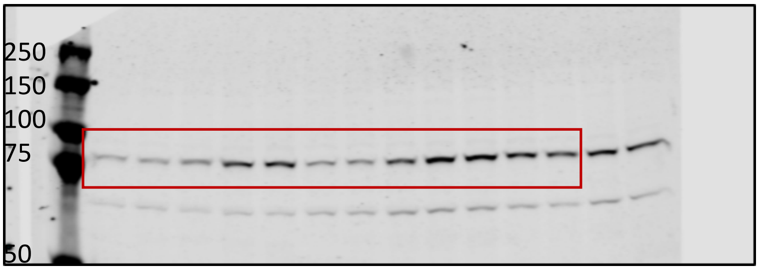

IB: α-pTyr705 STAT3

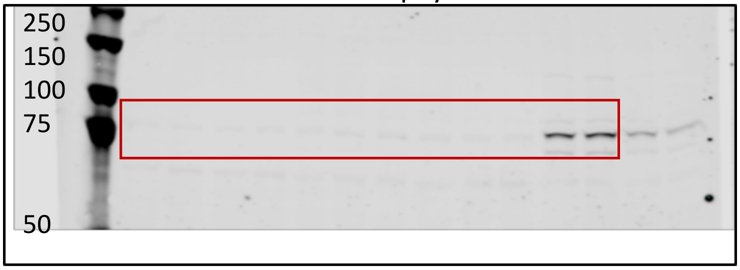

IB: α-STAT3

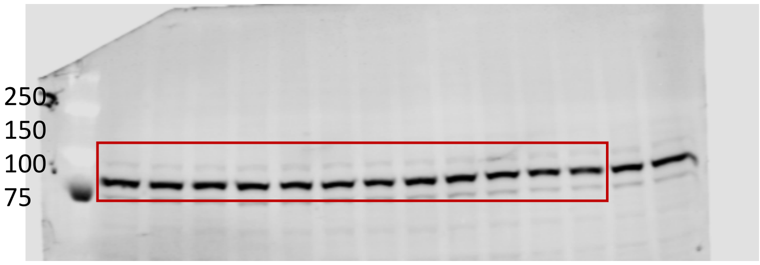

IB: α-TBK-1

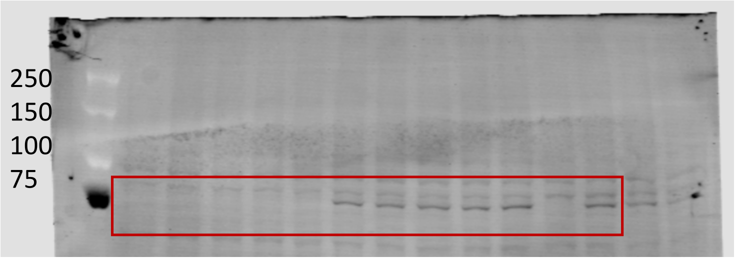

Uncropped immunoblots from Figure 3e

IB: α-pS727 STAT3

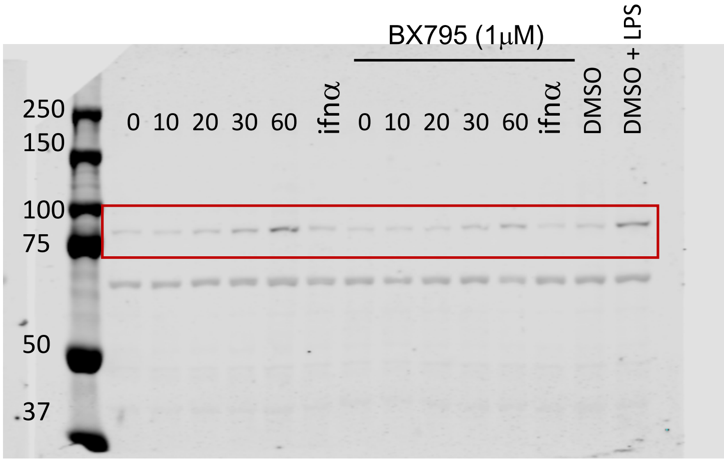

IB: α-STAT3

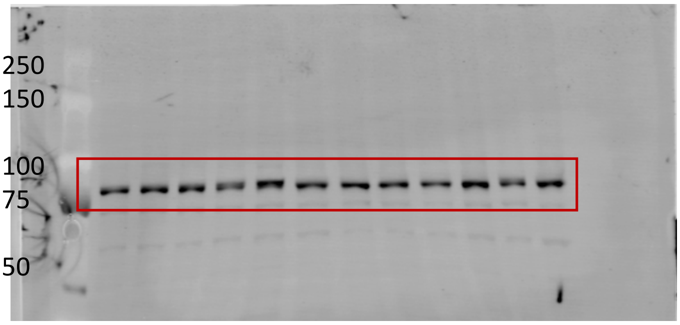

IB: α-pTBK-1

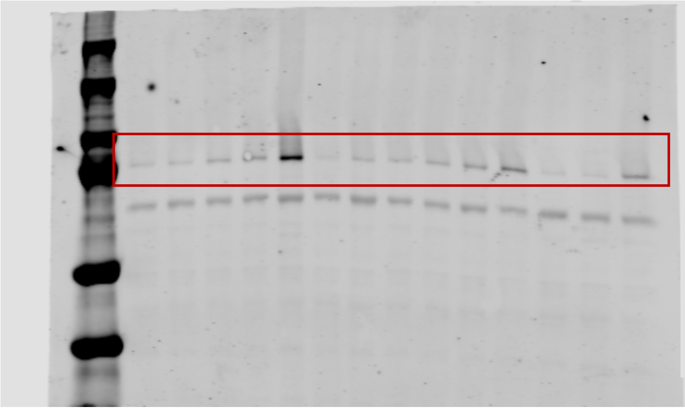

IB: α-TBK-1

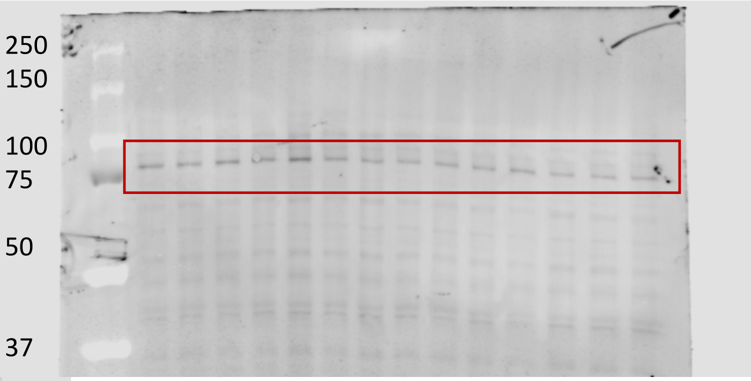

Uncropped immunoblots from Supplementary Figure 2

IB: α-pS727 STAT3

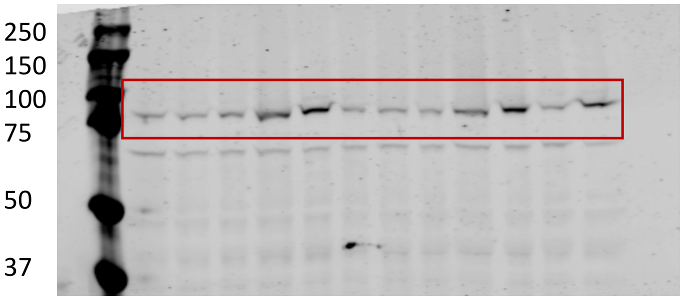

IB: α-STAT3

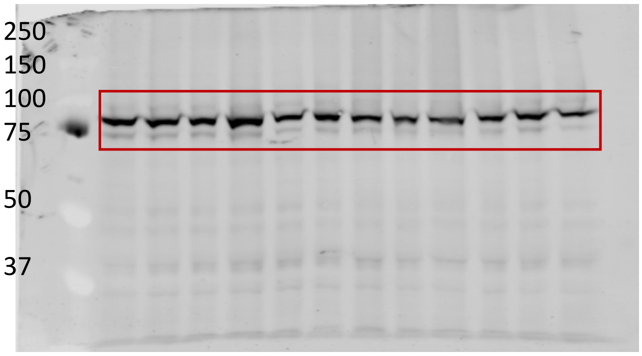

Supplement: Supplementary file 1 — Supplementary Information [file 41467_2020_17669_MOESM1_ESM.pdf]
