# Supplementary material for: STAT3 serine phosphorylation is required for TLR4 metabolic reprogramming and IL-1β expression
Source: Nat Commun. 2020 Jul 30;11:3816. doi: 10.1038/s41467-020-17669-5 (PMC7393113; doi:10.1038/s41467-020-17669-5)
Supplement: Supplementary file 3 — Descriptions of Additional Supplementary Files [file 41467_2020_17669_MOESM3_ESM.pdf]

Descriptions of Additional Supplementary Files

Supplementary Dataset 1

Description: Compound names and chemical identities of the library of 355 kinase inhibitors. List of the kinase inhibitors used to identify potential kinases involved in LPS-induced mitochondrial superoxide production (related to Figure 3a and 6 Supplementary Figure 1).

Supplementary Dataset 2

Description: Kinase inhibition of LPS-induced mtROS. iBMDMs were pretreated with kinase inhibitors (500nM) for 60 mins, and MitoSOX added 10 mins prior to challenge with LPS for 1, 2 or 4 h. Fluorescence intensity was measured at 580nm in a BMG ClarioStar as a readout of mitochondrial superoxide and presented as the fold increase of fluorescence intensity compared to unstimulated control (related to Supplementary Figure 1). Data is represented as the means of 3 biological replicates per inhibitor.

Supplementary Dataset 3

Description: Densitometry analysis of LPS-induced pS727 STAT3. BMDMs were treated or not with PI3K inhibitor TG10073 for 60 mins prior to LPS stimulation for indicated times. Results represent densitometry analysis of immunoblots of pS727 STAT3 compared to total STAT3 at each indicated timepoint as determined using ImageJ.
